# Maternal stress and vulnerability to depression: coping and maternal care strategies and its consequences on adolescent offspring

**DOI:** 10.1038/s41398-022-02220-5

**Published:** 2022-11-04

**Authors:** Renata L. Alves, Camila C. Portugal, Igor M. Lopes, Pedro Oliveira, Cecília J. Alves, Fernando Barbosa, Teresa Summavielle, Ana Magalhães

**Affiliations:** 1 Addiction Biology, i3S - Instituto de Investigação e Inovação em Saúde and Instituto de Biologia Molecular e Celular (IBMC), Universidade do Porto, Porto, Portugal; 2 Laboratory of Neuropsychophysiology, Faculty of Psychology and Education Sciences, University of Porto, Porto, Portugal; 3 Glial Cell Biology, i3S – Instituto de Investigação e Inovação em Saúde and Instituto de Biologia Molecular e Celular (IBMC), Universidade do Porto, Porto, Portugal; 4 Estudos de Populações, Instituto de Ciências Biomédicas Abel Salazar, Universidade do Porto, Porto, Portugal; 5 Neuro & Skeletal Circuits, i3S - Instituto de Investigação e Inovação em Saúde and Instituto Nacional de Engenharia Biomédica (INEB), Universidade do Porto, Porto, Portugal; 6 Departamento de Ciências Funcionais, Escola Superior de Saúde (ESS), Politécnico do Porto, Porto, Portugal; 7 Behavioural Sciences, Instituto de Ciências Biomédicas Abel Salazar, Universidade do Porto, Porto, Portugal; 8 Instituto Universitário de Ciências da Saúde, CESPU, CRL, 4585-116 Gandra, Portugal

**Keywords:** Learning and memory, Molecular neuroscience

## Abstract

Depressive mothers often find mother-child interaction to be challenging. Maternal stress may further impair mother-child attachment, which may increase the risk of negative developmental consequences. We used rats with different vulnerability to depressive-like behavior (Wistar and Kyoto) to investigate the impact of stress (maternal separation-MS) on maternal behavior and adolescent offspring cognition. MS in Kyoto dams increased pup-contact, resulting in higher oxytocin levels and lower anxiety-like behavior after weaning, while worsening their adolescent offspring cognitive behavior. Whereas MS in Wistar dams elicited higher quality of pup-directed behavior, increasing brain-derived neurotrophic factor (BDNF) in the offspring, which seems to have prevented a negative impact on cognition. Hypothalamic oxytocin seems to affect the salience of the social environment cues (negatively for Kyoto) leading to different coping strategies. Our findings highlight the importance of contextual and individual factors in the understanding of the oxytocin role in modulating maternal behavior and stress regulatory processes.

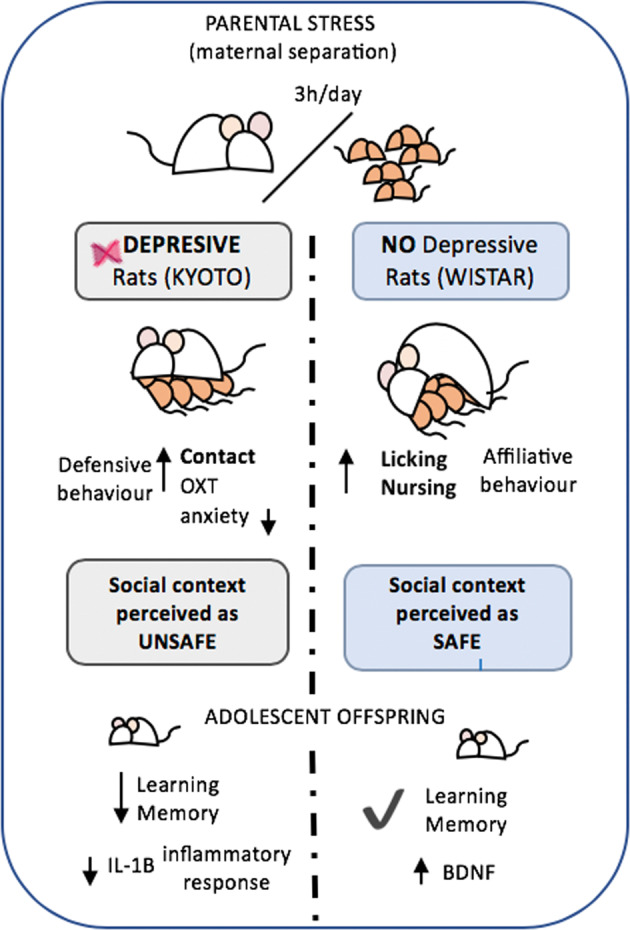

## Introduction

Depressive mothers often display higher difficulty in interacting with their child, increasing the liability of child abuse and negligence [1]. In addition, maternal adverse events cause increased maternal stress, which may translate to a higher rate of depressive mothers, and rise of the worldwide number of children growing up under chronic stress [2]. Thus, maternal stress may have long-lasting effects on mothers, their children, and their families. Despite the importance of vulnerability to depression and its relationship with maternal stress, there is a lack of research combining the impact of such risk factors on mother’s behavior and its consequences for the offspring.

In rodents, maternal behavior allows a proper mother/pup attachment, adequate nutrition, and temperature regulation [3]. It includes a complex variety of behaviors and its quality/quantity have been linked to the development of emotional and cognitive processes in the offspring [4]. Maternal behavior is regulated by oxytocin [5], a neuropeptide released from the paraventricular nucleus and the supraoptic nucleus of the hypothalamus [6]. While oxytocin activates brain areas related to maternal behavior, its blockage leads to maternal care deficits [7, 8]. Furthermore, the developing oxytocinergic system is impacted by mother’s absence [9]. The maternal separation paradigm (MS), used as a maternal adverse event, is the most widely used animal model to study early-life stress and disruption of the infant-mother relationship [10]. In a recent systematic review, we found that MS induces changes in dam’s emotional behavior by increasing anxiety and depressive-like symptoms [4], aggression, and impacting on maternal care [11]. MS seems to increase the total time spent in maternal behavior [4, 12], which was suggested to likely represent an attempt to overcompensate for the time off-nest [12]. Under MS, pups increase solicitation calls [13], compelling mothers to increase their maternal behavior as a response to the pup needs [4], which has been associated with increased expression of oxytocin receptors (OXTR) [14].

The existing literature seems to indicate that MS induces changes in the hippocampus of the offspring [15, 16], leading to a maladaptive brain development and cognitive impairment in adulthood [17]. The impact of maternal separation on the hypothalamic-pituitary-adrenal (HPA) axis is well-recognized [18, 19]. However, other factors are increasingly recognized as also relevant in triggering MS-induced behavioral alterations, like anxious and depressive-like behaviors [20]. Early-stress life events, including MS, have been associated with changes in immune activation and seem to affect microglia proliferation, morphology and phagocytic activity [21], priming them to contribute to later neuropsychiatric disorders [22]. The potential interplay between the immune system and depression, however, was yet barely investigated in MS models.

Psychosocial stressors can lead to increased microglial reactivity in the hippocampus, which may also contribute to cognitive impairment with neuroinflammation as a crucial mediator [23, 24]. Neuroinflammation is also linked to depression [25], primarily by its association with pro-inflammatory cytokines increased expression, such as the tumor necrosis factor (TNF), interleukin (IL)-6 and IL-1β [26–28].

In the present study, we used a depressive-like animal model (Wistar-Kyoto rat strain) combined with the MS paradigm to investigate the interplay between the role of pre-existing vulnerability to depression and environmental/social risk factors. In the present study, we used a depressive-like animal model (Wistar-Kyoto rat strain) combined with the MS paradigm to investigate the interplay between the role of a pre-existing vulnerability to depression and environmental/social risk factors. The Wistar-Kyoto (from now on referred as Kyoto) rat strain is a validated animal model of endogenous depression, which allows studying vulnerability to genetic depression [29]. The Kyoto strain was obtained by successive selective breeding resulting in rats that present: (i) excessive immobility in the forced swim test (FST), (ii) reduced activity in the open field (OF), (iii) increased anxiety-like behavior, (iv) behavioral despair, (v) lower social interaction and exploration, and (vi) anhedonia [30, 31]. Upon these behavioral characteristics, Kyoto display alterations in the HPA axis, characterized by decreased corticosterone (CORT)-mediated responses to acute stress and deficits in the serotonergic, dopaminergic and neurotrophic systems, thus exhibiting a behavioral and neurobiological phenotype with well-accepted face validity in depression [30, 32]. Emerging studies suggest also the involvement of neuroinflammation [31]. Thus, we studied the MS impact on maternal care and mother’s anxiety/depressive-like behaviors focusing on the oxytocinergic system and neuroinflammatory processes interactions. We predicted that depressive-like mothers, under maternal stress, would worsen their maternal behavior linked to a reduction in oxytocin. Furthermore, we expected that the mother’s maternal-stress-response impact on the adolescent offspring cognition would be worse in the depressive-like group and that it would be related to the pro-inflammatory cytokine levels.

## Methods

### Animals

**Fig. 1:**
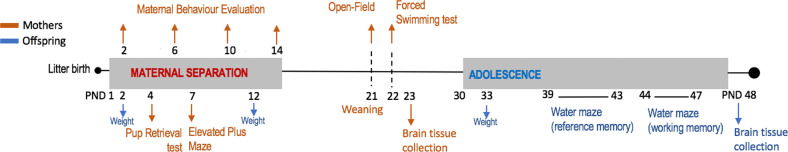
Schematic timeline summarizing the experimental procedures. PND = Postnatal day.

Wistar and Kyoto rats were obtained from Charles River (Barcelona/Spain). Rats were kept under standard conditions (21 ± 1 °C; 60 ± 5% humidity; 12h/12h light cycle – off at noon) with *ad libitum* food/water. Pregnant females were individually housed on pregnancy day 16. Animal behavior analyses were performed with the software Observer XT version10 (Noldus information technology, Netherlands) and Smart Video Tracking Software (Panlab) version 3.0, following the timeline in Fig. 1. Brain areas were collected 24 h after behavioral tests. All experiments involving animals were approved by the Portuguese regulatory agency Direcção Geral de Alimentação e Veterinária (DGAV) and the ethics committee of IBMC-i3S. The facility and people directly involved in animal experimentation were certified. All animal experiments followed the European guidelines for animal welfare (2010/63/ EU Directive).

### Maternal separation

Each litter was sexed and adjusted to 8–10 pups in PND1 (PND0 = delivery day). Dams from each strain were randomly assigned to control (*n* = 8/strain) or MS group (*n* = 7/strain). Control group followed animal facility rearing (1 cage change/week). The MS dams were separated from their offspring at 9:00 am, 180 min/day, from PND2-14, following the MS protocol described by Alves et al. [33]. This study used 88 pups and the remaining were used in a different study [33]. Rats were weaned and co-housed (2-3/cage) at PND21 and 8 groups were established (*n* = 11/group): control Wistar male; control Wistar female; control Kyoto male; control Kyoto female; MS Wistar male; MS Wistar female; MS Kyoto male; and MS Kyoto female. Each group contained rats from all litters. Pup’s weight was measured at PND2, 12, 33.

### Mothers evaluation

#### Maternal behavior

Maternal behavior toward offspring was scored immediately after pup reunion, at PND 2, 6, 10, 14, for 10 min. Animals were not disturbed during behavioral observations. The following behavior categories were observed: total maternal behavior (affiliative plus non-affiliative behavior); affiliative behavior (grooming/licking and nursing time); non-affiliative behavior (dam in contact with the offspring but not in grooming/licking and nursing).

#### Pup retrieval test (PRT)

At PND4, pups were returned to the original cage after MS away from the nest, followed by the return of the mother. Control group mothers were removed for 3 min and returned, while their pups were moved to the opposite-nest side. The observation ended when mothers retrieved all pups, or the 10 min observation limit was achieved [34]. First-contact latency; first-pup-retrieval latency; and full litter retrieval time were quantified.

#### Elevated plus maze (EPM)

Mother anxiety-like behavior was measured during the MS period (PND 7). The apparatus consisted of four connected arms (44 cm × 14 cm) in a plus-shaped structure and elevated 72 cm above the floor. Two arms were an open platform (no sidewalls) and two were enclosed within 22 cm-high sidewalls. Arms were positioned so opposing arms were of the same type. Each mother was placed in the central platform (the intersection of the four arms - 14 × 14 cm area) facing an open arm and left undisturbed for 5 min. Testing was conducted in the dark phase of the light/dark cycle. The apparatus was cleaned between animals with neutral, odor free soap [35]. The following behaviors were measured: (a) open-arms time; (b) open-arm entries; (c) center-area time; (d) immobility time; (e) total arm entries; (f) rearing; and (g) head dipping.

#### Open-field (OF)

On weaning day (PND21), mothers were tested in the OF to measure anxiety-like symptoms. The apparatus consisted of an empty rectangular arena (80 × 60 cm). Mothers were placed in the corner of the apparatus and left to explore the arena for 10 min. The following behaviors were recorded: (a) center time; (b) center entries; (c) center latency; (d) total distance traveled; and (e) rearing.

#### Forced swimming test (FST)

Mother passive coping behavior was assessed as a depressive-like behavior indicator with the FST at PND22 according to the protocol described by Aguggia et al. [34]. Dams were placed in a transparent container filled with water (25 ± 2 °C) for 10 min (pre-test session to reduce acute stress) and tested 24 h later for 5 min in the same container. Swimming, climbing, and immobility duration were recorded.

### Offspring evaluation

#### Morris water maze (MWM)

The MWM test was used to evaluate the impact of MS on the cognitive performance of the adolescent offspring. The apparatus consisted of a water-filled (24 °C) circular pool (111 cm diameter). The procedure was adapted from Vorhees and Williams [36]. In preparation for the memory task tests, cued learning was conducted at PND37-38. Four trials were performed during which both, start quadrant and platform locations were changed. If a rat did not locate the platform before 60 s elapsed, it was guided to the platform and could stay there for 15 s.

#### Spatial learning and reference memory

After cued learning, rats were subjected to the spatial learning task (PND39–42, four trials/day). Extra maze cues were placed in the room and the platform was submerged in a fixed location. Latency to reach the platform was recorded. On probe day (PND43), the reference memory was evaluated (1 trial). The platform was removed, and rats were released in the quadrant opposite the platform’s previous location. Rats could swim freely during 30 s. The following measures were quantified: (a) target mean distance; (b) total distance traveled; (c) target quadrant time percentage; (d) target quadrant latency; (e) target crossings.

#### Spatial working memory

At PND44–47, the platform and rat starting positions were the same in each day but changed every day (four trials/day, adapted from Tata et. al. [37]). Maze cues were kept as in the spatial learning task. Latency to reach the platform was recorded. Only latency to reach the platform within the day was expected to decrease, but not between days.

### Mothers and offspring biomarkers

#### Protein extract preparation

Dams were sacrificed at PND24, 24 h after the FST. The adolescent offspring were sacrificed at PND48, 24 h after the spatial working memory test.

For dams, the amygdala (Bregma −1.60 to −2.30) [38], the prefrontal cortex (PFC, Bregma 2.70 to 5.20) [38], and the whole hippocampus were homogenized in a buffer containing 50 mM Tris-Base, 10 mM EDTA, 150 mM NaCl, 0.1% Tween 20 and protease inhibitors [39]. The offspring’s hippocampus was homogenized using the same buffer. Samples were sonicated and centrifuged for 15 min at 2271 × *g* and posteriorly, the supernatant was centrifuged for 5 min at 13500 g. The protein concentration was estimated using Pierce BCA Protein Assay kit (Thermo Scientific), following the manufacturer’s instructions. After centrifugation, total protein concentration was determined in the supernatant using a BCA kit (23227, ThermoFisher Scientific).

#### Enzyme-linked immunosorbent assays (ELISA)

Oxytocin was measured by ELISA (ADI-900-153A, Enzo Life Sciences – Detection range: 15.6 to 1.000 pg/ml) in mother’s hypothalamus protein homogenates obtained by dissecting the hypothalamic region between Bregma −0.26 and −1.40 [38], which encompass the medial preoptic area (MPOA), the paraventricular and supraoptic (suprachiasmatic) nuclei. BDNF (mediator of neuronal plasticity [40]) levels were measured in offspring’s hippocampus homogenates by ELISA (CYT306, Millipore). The quantification of IL‐1β, IL-6, and TNF in the mother’s hippocampus and of IL-1β and IL-6 in the offspring’s hippocampus were also performed by ELISA (900-M91, 900-M86, 900-M73, PeproTech).

#### Western blot

We determined the expression of (i) OXTR on mother’s PFC, hippocampus (100 µg protein), and amygdala (70 µg protein); (ii) postsynaptic density protein 95 (PSD95, abundant scaffold protein present in the glutamatergic postsynaptic terminal [41]) and vesicular glutamate transporter 1 (vGlut1, a presynaptic transporter protein that mediates glutamate uptake into synaptic vesicles, linked to the efficacy of glutamatergic neurotransmission [42]) on offspring’s hippocampus (using 30 µg protein). Samples were denatured at 95 °C for 5 min and loaded into a 10% SDS-page gel, transferred to a nitrocellulose membrane, processed with blocking solution, and incubated with primary antibody overnight, at 4 °C (OXTR, 1:400 (Thermo Fisher Scientific); PSD95, 1:2000 (ThermoFisher Scientific); vGlut1, 1:1000 (Synaptic Systems); GAPDH, 1:100,000 (HyTest). Primary antibodies were visualized with HRP-conjugated anti-rabbit or HRP-conjugated anti-mouse (1:10,000, Jackson ImmunoResearch). Signals were enhanced using SuperSignal™ West Pico PLUS Chemiluminescent Substrate (ThermoFisher Scientific). Data was quantified using ImageJ software [43].

### Statistical analyses

Sample size was determined based on literature and previous experience that used the same strain and behavioral tests used in this study. Mother rats and their offspring were allocated to experimental groups randomly. Since treatment (maternal separation vs control) had to be applied to whole offspring, litter effect was considered random effect during analysis. Animals from the same litter were later divided into different tests. The investigator was not blinded to the group allocation but had not control over the animal testing order.

Normality and homoscedasticity were verified (Shapiro–Wilk or Kolmogorov–Smirnov test and Levene’s test respectively). Treatment effects on dams were determined with two-way ANOVA (Strain: Wistar/Kyoto, and MS: control/MS). Generalized linear models with 3 factors (Strain: Wistar/Kyoto MS: control/MS, and Sex: male/female) and their interactions were used for the offspring analyses with normal or Poisson distributions to obtain normal distribution of the model residuals and homoscedasticity. Time was added as a fourth factor for learning and working memory results. A Linear Mixed Model was also used to verify litter random effect, but significance was not found (except for pup weight). Multiple comparisons were Bonferroni adjusted (dams) or Tukey-Kramer adjusted (offspring). Data transformation, when needed due to heteroscedasticity, was: arcsine of percentages; square-root of zero containing data, and log transformation. IBM SPSS Statistics 23 software was used and SAS OnDemand for Academics software. Copyright © 2021 SAS Institute Inc, Cary, NC, USA. *α* = 0.05.

## Results

### Mothers

#### Wistar dams presented higher-quality maternal behavior than Kyoto dams in response to MS

**Fig. 2:**
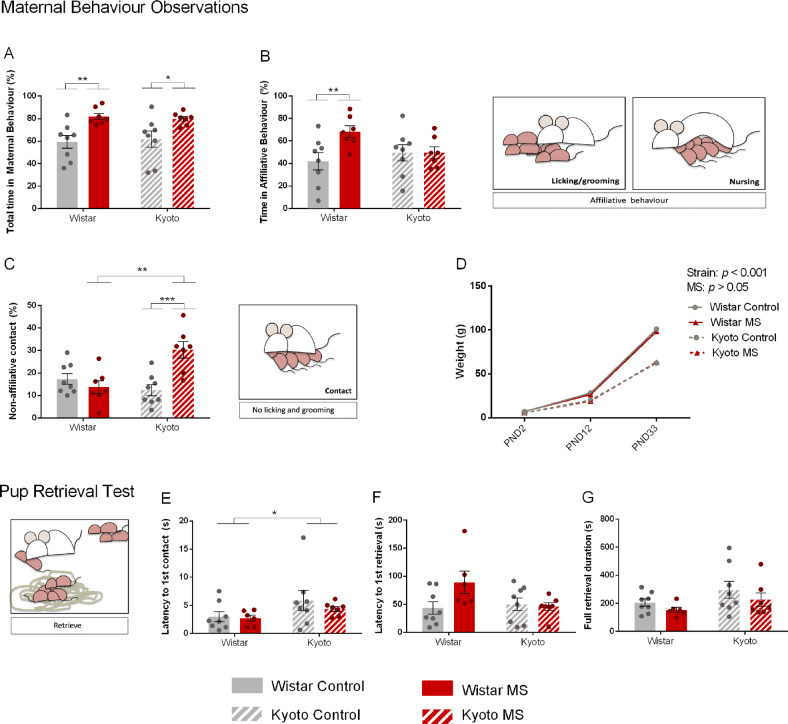
Maternal separation impacts maternal behavior of mothers with different vulnerabilities to depression. **A** MS increased maternal behavior in both strains. However, Wistar MS dams increased **B** affiliative behavior (high-quality behaviors) and Kyoto MS dams increased **C** non-affiliative contact time. **D** Wistar showed higher weight gain than Kyoto strain, but it was not impacted by MS. Data were analyzed by two-way ANOVA (2 × 2 factorial design) with Bonferroni comparisons, except for **D** which was analyzed with a linear mixed model (strain and MS as fixed effects and litter as random effect). **E** Latency to first contact was higher in the Kyoto strain. **A**, **B**, and **C** were data arcsine transformed. **E**, **F**, and **G** data were log-transformed. *n* = 8 (controls Wistar and Kyoto); *n* = 7 (MS Wistar and Kyoto, except for PRT: *n* = 6 for MS Wistar). Results are expressed as mean ± SEM. *p < 0.05, **p < 0.01, ***p < 0.001. MS = maternal separation; PND = postnatal day.

MS impact on mothers and their offspring occurs due to the separation period but more importantly, due to changes in their interaction afterward. Thus, we started by investigating the MS effect on maternal behavior. Total maternal behavior was higher in both MS groups (*F*_(1,26)_ = 15.7, *p* ≤ 0.001). MS dams spent more time in maternal behavior than control dams of the same strain (Wistar: *p* < 0.01; Kyoto: *p* < 0.05) (Fig. 2A). Within Wistar dams, affiliative behavior was higher in MS group (*p* < 0.01), but not for Kyoto (Fig. 2B). In contrast, MS Kyoto dams had more non-affiliative contact than their control (*p* < 0.001) and MS Wistar (*p* < 0.001) (Fig. 2C). The MS impact on non-affiliative contact was strain-dependent (Strain X MS: *F*_(1,26)_ = 14.0, *p* ≤ 0.001; MS: *F*_(1,26)_ = 4.95, *p* < 0.05). Pup weight was only affected by strain (*p* < 0.001) (Fig. 2D). We also performed the Pup Retrieval Test (PRT), revealing that Kyoto dams present a higher latency to the first mother/pup contact (Strain: *F*_(1,25)_ = 4.55, *p* < 0.05) (Fig. 2E). However, the first retrieval latency and the total retrieval time had no differences (Fig. 2F, G). Taken together, control Wistar and Kyoto dams spent similar time in maternal behavior. However, under MS conditions, the strains increased differently the time spent in maternal behavior, with higher affiliative behavior for Wistar and higher non-affiliative contact for Kyoto.

#### Increased exploration in Wistar dams during the MS period and decreased anxiety-like behavior in Kyoto dams after the weaning period

**Fig. 3:**
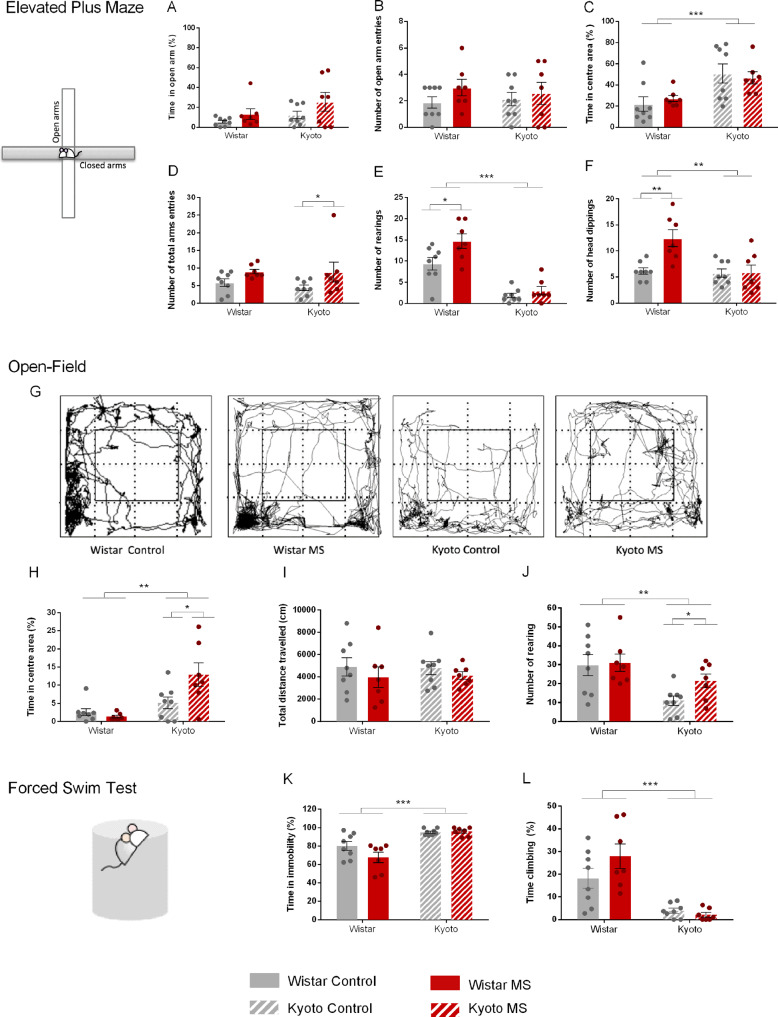
Maternal separation affects mothers’ emotional behavior. During MS period (using EPM), **A**, **B** no differences were found regarding anxiety. MS increased **D** Kyoto total arm entries and increased Wistar **E** number of rearing and **F** number of head dipping. **G** example of Wistar and Kyoto control groups tracking in OF (Smart v3.0, Panlab, Barcelona, Spain; https://www.panlab.com/en/). After weaning (using OF), MS increased Kyoto **H** time in center area and **J** number of rearing, indicating decreased anxiety levels. FST confirmed Kyoto depressive profile since Kyoto strain showed higher time **K** immobility and lower **L** climbing comparing with Wistar. MS did not impact depression-like behaviors. Data were analyzed by two-way ANOVA (2 × 2 factorial design) with Bonferroni comparations. **A**, **C**, **H**, **K**, **L** Data were arcsine transformed. **B**, **D**, **E**, **F** Data were log-transformed. **I**, **J** Data were square-root transformed. *n* = 8 (control and MS Wistar); *n* = 7 (control and MS Kyoto). Results are expressed as mean ± SEM. *p < 0.05, **p < 0.01, ***p < 0.001. MS = maternal separation.

We hypothesize that an animal that experienced maternal-parental stress is expected to alter its emotional state, and thus, mothers’ exploratory and anxiety-like behaviors were also assessed. The EPM was conducted during the MS protocol period while the OF was conducted after weaning. In the EPM, no strain or MS differences were found for the time or entries in the open arms (Fig. 3A, B). However, Kyoto dams spent more time in the EPM center than Wistar, independently of MS (Strain: *Wald χ*^*2*^ = 16.6, *p* < 0.001) (Fig. 3C) and that the Kyoto increased the total arm entries due to MS (MS: *F*_(1,26)_ = 7.55, *p* < 0.05) (Fig. 3D). In contrast, Wistar dams displayed more rearing (Strain: *F*_(1,26)_ = 50.4, *p* ≤ 0.001*, MS: F*_(1,26)_ = 4.22, *p* ≤ 0.05), with MS further increasing the Wistar rearing (*p* < 0.05) (Fig. 3E). Head dipping followed the same pattern, with Wistar dams performing more head dipping (Strain X MS: *Wald χ*^*2*^ = 7.32, *p* < 0.01; Strain: *Wald χ*^*2*^ = 10.2, *p* ≤ 0.001; MS: *Wald χ*^*2*^ = 6.08, *p* < 0.05) and MS impacting head dipping only in Wistar (*p* < 0.001) (Fig. 3F).

After weaning, exploratory and anxiety-like behaviors were studied using the OF (Fig. 3G). Kyoto dams spent more time in the center (Strain X MS: *F*_(1,26)_ = 5.07, *p* < 0.05; Strain: *F*_(1,26)_ = 12.1, *p* < 0.01), with MS Kyoto dams spending more time in the center area than the control Kyoto (*p* < 0.05) and MS Wistar dams (*p* ≤ 0.001) (Fig. 3H). Total distance was similar in all groups (Fig. 3I) and the exploratory behavior, assessed as rearing occurrence, was different between the two strains (Strain: *F*_(1,26)_ = 11.4, *p* < 0.01). Kyoto dams displayed fewer rearing, yet MS Kyoto dams performed more rearing than their control (*p* < 0.05) (Fig. 3J).

Altogether, the results obtained in the EPM suggest that anxiety-like behavior was not affected during the MS protocol. However, Wistar dams displayed more exploratory behaviors than Kyoto dams and MS further increased exploration in Wistar. The OF results suggest that Kyoto dams present less anxiety-like behavior after weaning than the Wistar and MS resulted in less anxiety-like behavior in the Kyoto dams. Although MS increased exploration only in Kyoto, Wistar dams displayed more exploratory behavior.

#### MS did not influence depressive-like behavior in dams

As expected due to their strain characteristics, Kyoto dams spent more time immobile in comparison with Wistar dams in the FST (*F*_(1,26)_ = 36.3, *p* < 0.001) (Fig. 3K). Consistently, Kyoto dams also spent less time climbing (*F*_(1,26)_ = 43.5, *p* < 0.001) (Fig. 3L). Kyoto dams presented more depressive-like behaviors than Wistar, with no observed effect from MS, which may be due to a ceiling effect since the Kyoto control already spent most of the test immobile.

#### MS increased oxytocin expression in the hypothalamus of Kyoto dams

**Fig. 4:**
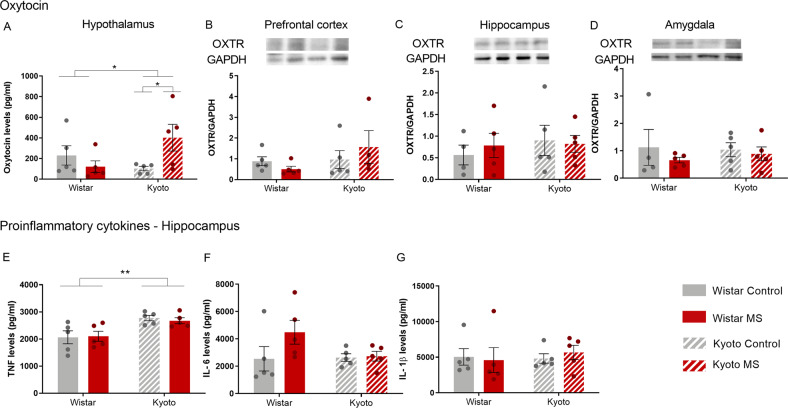
Maternal separation disturbed mothers’ neurobehavior. After weaning, MS increased Kyoto **A** oxytocin levels, in agreement with the decrease of anxiety observed. **E** Kyoto showed high levels of TNF than Wistar strain. Quantification of **A** oxytocin (pg/ml) levels in hypothalamus by ELISA; OXTR in **B** PFC, **C** hippocampus, and **D** amygdala by Western blotting; **E** TNF (pg/ml), **F** IL-6 (pg/ml), and (**G**) IL-1β (pg/ml) levels in hippocampus by ELISA. Data were analyzed by two-way ANOVA (2 × 2 factorial design) with Bonferroni comparisons. **A**–**C**, **F**, **G** data were log-transformed. *n* = 5, except for **B** MS Kyoto, and **C**, **D** for control Wistar: *n* = 4. Results are expressed as mean ± SEM. *p < 0.05, **p < 0.01, ***p < 0.001. MS = maternal separation.

Since maternal behavior is strongly influenced by oxytocin, we analyzed the expression of oxytocin in the hypothalamus of dams. Surprisingly, MS Kyoto dams presented higher oxytocin expression (Strain X MS: *F*_(1,16)_ = 6.60, *p* < 0.05) (Fig. 4A). Because altered levels of oxytocin may result in altered expression of OXTR in oxytocin projection regions, we evaluated the expression of this receptor in oxytocin target regions that are recognized as relevant for regulation of maternal behavior, such as the PFC, the hippocampus, and the amygdala. No differences were observed in the OXTR expression (Fig. 4B–D).

#### Kyoto dams present higher TNF in the hippocampus than Wistar dams

As pro-inflammatory cytokines can influence anxiety- and depressive-like behaviors, we analyzed the expression of TNF, IL-6, and IL-1β in the hippocampus. Kyoto dams presented higher expression of TNF (*Wald χ*^*2*^ = 18.3, *p* < 0.001) (Fig. 4E). Concerning IL-6 (Fig. 4F) and IL-1β (Fig. 4G) no significant differences were observed.

### Offspring

#### MS worsened the spatial learning and the reference memory in Kyoto adolescents

**Fig. 5:**
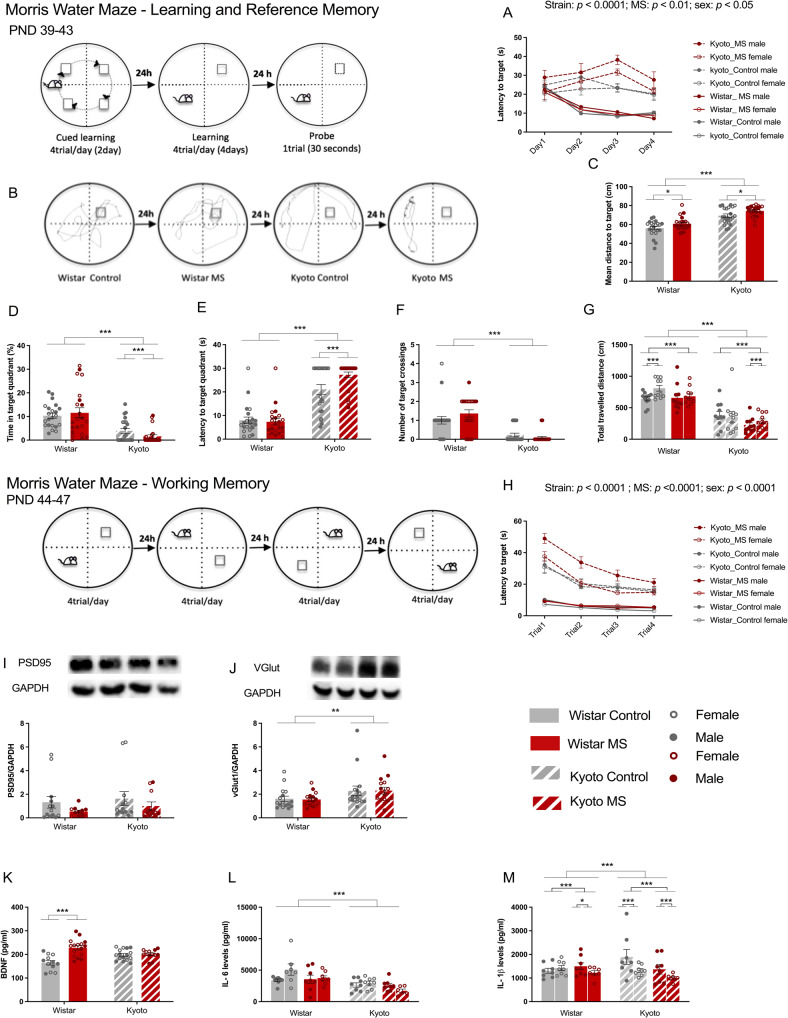
Maternal separation worsened Kyoto adolescent offspring’s cognitive performance. MS increased Kyoto **A** latency to reach the platform, showing learning impairment. Regarding probe day (**B**–**G**) MS slightly affected both strains. **H** MS-induced poor performance in working memory. **J** VGlut expression was higher in Kyoto strain. **K** MS led to an increase of BDNF level on Wistar, which may be related to the increase of affiliative behavior received by their mothers. **M** MS altered IL1-β levels on Kyoto that can be associated with cognitive impairments presented. Quantification of **I** PSD95 and **J** VGlut in hippocampus by Western blotting; **K** BDNF (pg/ml), **L** IL-6 (pg/ml), and **M** IL-1β (pg/ml) levels in hippocampus by ELISA. Generalized linear models with 3 factors (strain: Wistar/Kyoto, MS: control/MS, and sex: male/female) and their interactions were used. Time was added as a fourth factor for learning and working memory results. **A**, **F**–**M** used models with normal distribution and link function = identity, **C**–**E** used Poisson distribution with link function = log. **A**, **C**–**G** *n* = 22. **I**–**M** *n* = 16, except for **I** *n* = 13 for control Wistar and Kyoto, *n* = 11 for MS Wistar and Kyoto; **K** *n* = 12 for control Wistar and MS Kyoto, **L** *n* = 15 for control Wistar and *n* = 14 for MS Kyoto, **M** *n* = 15 for MS Kyoto. Results are expressed as mean ± SEM. *p < 0.05, **p < 0.01, ***p < 0.001. MS = maternal separation; PND = postnatal day.

We evaluated the offspring cognitive ability using the MWM. Kyoto adolescents displayed reduced spatial learning when compared to the Wistar group (represented by a reduction in latency over the learning days) (Fig. 5A). MS Kyoto performed poorly than the Kyoto, with MS Kyoto female adolescents presenting longer latency to the target (Strain: *Chi-Square*_*(1)*_ *=* 157.26, *p* < 0.0001, MS:*Chi-Square*_*(1)*_ *=* 10.12, *p* < 0.01, Strain X MS:*Chi-Square*_*(1)*_ *=*11.59, *p* < 0.001, Sex: *Chi-Square*_*(1)*_ *=* 6.11, *p* < 0.05, Strain X Sex: *Chi-Square*_*(1)*_ *=* 5.2*8*, *p* < 0.05, Strain X Days:*Chi-Square*_*(1)*_ *=* 57.78, *p* < 0.0001) (Fig. 5A). During the probe test Kyoto performed poorly than Wistar, and a main sex effect was only observed for the total traveled distance (Fig. 5C–G). The mean distance to the target was higher for the Kyoto strain and MS worsened both strains (Strain, *Chi-Square*_*(1)*_ = 60.85, *p* < 0.0001; MS, *Chi-Square*_*(1)*_ = 7.74, *p* < 0.01) (Fig. 5C). Kyoto adolescents also spent less time in the target quadrant (strain, *Chi-Square*_*(1)*_ *=* 246.00, *p* < 0.0001), with the MS Kyoto group spending even less time in that quadrant (Strain X MS *Chi-Square*_*(1)*_ *=*20.64, *p* < 0.0001; *z* value = 4.19, *p* < 0.0001) (Fig. 5D). Regarding the latency to enter the target quadrant, Kyoto presented higher latency to enter it (*Chi-Square*_*(1)*_ *=* 387.79, *p* < 0.0001), with the MS Kyoto showing even higher latency (Strain X MS *Chi-Square*_*(1)*_ *=*7.81, *p* < 0.01; *z* value = −4.16, *p* < 0.0001) (Fig. 5E). A strain effect was also observed for target crossings, which were decreased in Kyoto adolescents (*Chi-Square*_*(1)*_ *=*38.72, *p* < 0.0001) (Fig. 5F). For total traveled distance we found main effects of strain, MS and sex, plus interactions between them (Strain: *Chi-Square*_*(1)*_ *=*6772.47, *p* < 0.0001, MS:*Chi-Square*_*(1)*_ *=*458.24, *p* < 0.0001, Strain X MS: Chi-*Square*_*(1)*_ = 204.40, *p*< 0.0001, Sex: *Chi-Square*_*(1)*_ *=*105.69, *p* < 0.0001, Strain X Sex: *Chi-Square*_*(1)*_ *=*6.64, *p* < 0.01, MS X Sex: *Chi-Square*_*(1)*_ = 5.55, *p* < 0.05; Strain X MS X Sex *Chi-Square*_*(1)*_ *=* 135.54, *p* < 0.0001), with the Kyoto strain traveling a shorter distance, and with MS groups traveling less then their respective controls (Wistar *vs* MS Wistar, *z* value = 6.41, *p* < 0.0001; Kyoto *vs* MS Kyoto, *z* value = 21,26, *p* < 0.0001). Upon that, we also found sex differences within both strains (Male Wistar *vs* Female Wistar, *z* value = −11,69, *p* < 0.0001; Male Kyoto *vs* Female Kyoto, *z*value = −4.60, *p* < 0.0001), and between MS within each strain, with males traveling less than females (Control Male Wistar *vs* Control Female Wistar, *z* value = −14,48, *p* < 0.0001; MS Male Wistar *vs* MS Female Wistar, *z* value = *−*2,26*, p* = 0.3138; Control Male Kyoto *vs* Control Female Kyoto, *z* value = −2.95, *p* = 0.0634; MS Male Kyoto *vs* MS Female Kyoto, *z* value = −8.42, *p* < 0.0001) (Fig. 5G).

Under the work memory protocol, the Kyoto presented a worse performance, with MS-induced changes resulting in an even poor performance (Strain: *Chi-Square*_*(1)*_ *=* 357.68, *p* < 0.0001, MS: *Chi-Square*_*(1)*_ *=* 21.47, *p* < 0.0001, *Trial Chi-Square*_*(1)*_ *=* 146.64, *p* < 0.0001; Strain X MS: *Chi-Square*_*(1)*_ *=* 11.81, *p* < 0.001; Strain X Trial: *Chi-Square*_*(1)*_ *=* 68.71, *p* < 0.0001; Strain X MS X Trial: *Chi-Square*_*(1)*_ *=* 8.72, *p* < 0.05) (Fig. 5H). For this test we also found sex differences for the Kyoto strain, where MS males performed better then MS females, independently of the trial (Sex, *Chi-Square*_*(1)*_ *=* 14.93, *p* < 0.0001, Strain X Sex: *Chi-Square*_*(1)*_ *=* 8.40, *p* < 0.01, MS X Sex; *Chi-Square*_*(1)*_ *=* 9.39, *p* < 0.01; Strain X MS X Sex, *Chi-Square*_*(1)*_ *=* 20.36, *p* < 0.0001; MS Male Kyoto *vs* MS Female Kyoto, *z* value = 7.24, *p* < 0.0001*)* (Fig. 5H).

Altogether, we observed that Kyoto adolescents presented reduced spatial learning, reference and working memory in comparison with age-matched Wistar, with MS worsening their performance, particularly in females.

#### Kyoto adolescents presented higher levels of vGlut1 expression

To better understand the observed learning and memory deficits, we analyzed the expression of two different glutamatergic synaptic markers the postsynaptic density protein 95 (PSD95), which is a well-characterized and abundant scaffold protein present in the glutamatergic postsynaptic terminal, and the vesicular glutamate transporter 1 (vGlut1) that is a presynaptic transporter protein, mediating glutamate uptake into synaptic vesicles, the expression levels of these transporter determines the vesicle load in glutamate, shaping the efficacy of glutamatergic neurotransmission.

For PSD95 hippocampal expression levels, no differences between strains, or resulting from MS were observed (Fig. 5I). Regarding the expression levels of vGlut1, we found only strain-related differences, with Kyoto adolescents presenting increased vGlut1 levels (Strain, *Chi-Square*_*(1)*_ *=* *4.23, p* < *0.05)* (Fig. 5J). These changes in vGlut1 may be associated with strain differences reported for the MWM assessments.

#### MS increased brain-derived neurotrophic factor (BDNF) expression in Wistar but not in Kyoto adolescents

BDNF is a crucial mediator of neuronal plasticity and a key factor for synapse formation/maintenance during development, as well as for memory processes. BDNF expression was impacted by MS (*Chi-Square*_*(1)*_ *=* 16.26, *p* < 0.0001) and an interaction between Strain and MS (*Chi-Square*_*(1)*_ *=* 14.37, *p* < 0.001), which translates in MS-induced increase in BDNF in Wistar adolescents when compared with their control (*z*-value = −5.93, *p* < 0.001) (Fig. 5K). This BDNF increase was previously linked to early aversive experiences that may trigger adaptive processes, allowing better adaptation to later events [44]. However, it is also possible that the increase in BDNF may result from improved maternal care (Fig. 2).

#### MS decreased IL-1β expression in Kyoto adolescents

Because BDNF levels may associate with altered inflammatory markers, and pro-inflammatory cytokines can influence learning, memory and shape the neuronal circuit, we decided to evaluate IL-6 and IL-1β expression. Kyoto adolescents presented lower IL-6 levels (strain: *Chi-Square*_*(1)*_ *=* 19.44, *p* < 0.0001), which were further decreased by MS in females (MS X Sex: *Chi-Square*_*(1)*_ *=* *4.65, p* < *0.05*, *z*-value = 2.88, *p* < 0.05*)* (Fig. 5L). For IL-1β, we found differences due to strain, MS and sex M (Strain, *Chi-Square*_*(1)*_ *=* 28.35, *p* < 0.0001; MS, *Chi-Square*_*(1)*_ *=* 278.01, *p* < 0.0001; strain X MS, *Chi-Square*_*(1)*_ *=* 751.09, *p* < 0.0001; Sex, *Chi-Square*_*(1)*_ *=* 546.10, *p* < 0.0001; Strain X Sex, *Chi-Square*_*(1)*_ *=* 671.26, *p* < 0.0001, MS X Sex, *Chi-Square*_*(1)*_ *=* 8.59, *p* < 0.01; Strain X MS X Sex, *Chi-Square*_*(1)*_ *=* 39.76, *p* < 0.0001) (Fig. 5M), with MS having opposite effects in Wistar, where MS increases IL-1β, and Kyoto, where MS decreases it. In addition, when considering sex differences, MS male Wistar presented higher IL-1β than females (MS Male Wistar *vs* MS Female Wistar, *z* value = 3.52, *p* < 0.05) (Fig. 5M). Likewise, in the Kyoto group, males also presented higher IL-1β with or without MS *(*Control Male Kyoto *vs* Control Female Kyoto, *z* value = *28.36, p* < 0.0001; MS Male Kyoto *vs* MS Female Kyoto, *z* value = 20,47, *p* < 0.0001) (Fig. 5M). These suggest that a worse cognitive performance may associated with lower levels of pro-inflammatory markers. Previous studies have determined the association between hippocampal-dependent learning deficits and reduced IL-1β [45].

## Discussion and conclusion

This study explored the impact of MS on the maternal behavior of dams with different vulnerabilities to depressive-like behavior and explored its consequences on adolescent cognitive performance. Considering the high comorbidity between mothers´ depression and low maternal care [1], one could predicted that MS would reduce maternal behavior in depressive-like Kyoto mothers. However, confirming the results reported in publications that reviewed this issue [4, 46], we observed an increase in maternal behavior, in both strains, in response to MS. Interestingly, each strain displayed different strategies for handling maternal stress: depressive-like mothers (Kyoto) spent more time in contact (simple physical contact) with pups, whereas non-depressive mothers (Wistar) spent more time directing their behavior towards pups, performing more affiliative behaviors (licking, grooming and nursing). These affiliative behaviors are the most common pup-oriented maternal behaviors [12] and they crucial for mother-pup attachment [47]. In contrast, during simple physical contact (skin contact), mothers lie over their pups, but without offspring-oriented behavior. The increased contact of Kyoto dams with offspring suggests that the mother keeps the offspring close, while keeping a watch on the environment around her, likely because she does not know when the pups will be taken from her again. This behavior in depressive-like rat dams seems to be a form of maternal defense to protect pups from unpredictable stress (MS). In fact, contact behavior was previously reported as an alternative maternal defense behavior towards pups when a threat was present [48]. These differences in maternal behavior seem to be due to distinct maternal experiences and/or distinct internal states of the dams. Our results showed that maternal depressive-like traits played an important role regarding how each dam adjusted its maternal behavior in response to maternal stress. In line with these results, a recent study, in humans, showed that mothers with personality disordered traits do not perceive themselves as having bonding impairments with their infants, although they are less sensitive during interactions [49]. This highlights the relevance of genetic components in the expression of maternal behavior, but further studies are needed to elucidate gene-environment interactions in depression/maternal behavior.

Of note, MS-related differences in maternal behavior did not impact pups’ weight gain (a measure of growth efficiency), which further supports a previous study that did not find differences between Wistar and Kyoto offspring’s physical development after MS [50]. It was also reported that offspring of low-licking mothers do not differ in weaning weight and survival rate [51]. Thus, even though the Kyoto maternal behavior appears to lack quality, these mothers exhibit awareness towards their pups and do not compromise the offspring’s physical health.

Maternal care variations are associated with differences in oxytocin and OXTR expression in the brain [52]. However, we did not find changes in OXTR in the analyzed regions, but we showed, for the first time, that when a depressive-like dam is exposed to maternal stress (MS), the hypothalamic expression of oxytocin is augmented. In accordance, previous findings reported that skin contact between mother and pups, but not maternal licking or grooming, was positively related to pups’ hypothalamic oxytocin [53, 54]. A similar mechanism can be happening in mothers and help explaining our observations. Oxytocin release seems to promote social cohesion in the mother-offspring dyad, serving as a defensive behavior toward potential and unpredictable threats. Considering the social salience hypothesis of oxytocin [55], the oxytocin increase in depressive mothers could enhance social sensibility [56] to negative social cues (offspring absence), eliciting a more protective behavior from depressive mothers, as a defense against a potentially dangerous intruder. The mechanism by which oxytocin affects social behavior depends both on contextual and individual factors [57], which seems to influence the sensitivity and interpretation of the emotional meaning or relevance of an event.

In the present study, MS Kyoto dams, in combination with higher levels of hypothalamic oxytocin, displayed decreased anxiety-like behavior decrease in the OF and increased arm entries in the EPM, suggesting exploratory behavior disinhibition, likely because of lower anxiety. This outcome supports studies reporting an oxytocin anxiolytic effect [58, 59] and an oxytocin increase in response to conditioned fear and restrain [60, 61]. In agreement, Light and colleagues (2004) [62] suggested that oxytocin release during stressful situations alleviates physiological stress. The decreased anxiety-like behavior observed in depressive-like mothers can be a result of increased oxytocin. Of note, according to a recent publication, that reported that more resilient rats (as the Wistar strain) no longer display FST effects in corticosterone, BDNF or IL-1β levels, 24 h after the test, while more susceptible rats (such as the Kyoto strain) still display increased corticosterone and IL-1β at the same time point, is also possible that the FST performed before dams’ sacrifice, may somehow contribute to differences in oxytocin [63].

Data from human studies support that, under great psychosocial stress, mothers with higher oxytocin levels present fewer anxiety symptoms, suggesting that oxytocin may protect women in stressful situations from developing major depression [64, 65]. Moreover, human studies share a common assumption that mothers with higher oxytocin levels present fewer depressive symptoms, compared to mothers with lower oxytocin levels, and this supports a model where low oxytocin levels represent a causal effect of Postpartum Depression (PPD) rather than a consequence [66]. However, there are also inconsistent findings in this issue, that may be due to discrepancies in sample size, differences in assessment timepoints and in the methodologies used to measure both oxytocin and depressive symptoms. Our and other studies [67] are beginning to shed light on the complex nature of oxytocin’s effect in depression, bringing a new perspective in which the oxytocin’s role is dependent on the individual experience, such as lack of social support and teenage parenting, or single parenthood. In this context, the role of oxytocin on PPD needs further investigation.

Wistar mothers increased licking, grooming, and nursing behaviors due to MS, with no impact on the oxytocinergic system. These results suggest that Wistar dams increased pro-social behavior to counteract MS effects, exhibiting a more positive emotional regulation in coping with maternal stress. These dams did not reveal changes in anxiety-like behavior due to MS, but interestingly increased exploration (represented by rearing and head dipping). Moreover, the lack of increased oxytocin expression in the hypothalamus of MS Wistar mothers corroborates the idea that Wistar mothers were more emotionally regulated, given that increased oxytocin levels in the hypothalamus are part of the physiological response to social stress [62]. Of note, attenuation of the anxiety response by oxytocin occurs in individuals with poor coping, but not in individuals with adequate coping [68, 69], which is in accordance with our observations for the Kyoto and Wistar strains.

In the absence of other comorbidities, depressive disorders are related with increased levels of in central and peripheral pro-inflammatory cytokines, particularly with TNF [26]. In agreement, TNF was higher in Kyoto dams than in Wistar. Studies report that oxytocin reduces TNF production, inducing an anti-inflammatory state, however, the mechanisms involved are elusive and in vitro results seem to indicate that oxytocin does not directly regulate TNF [70].

Maternal care critically affects offspring’s brain maturation and cognitive and emotional behaviors development in mammalian species [71]. We observed that depressive-like adolescents (Kyoto) had significant learning and memory deficits. MS had higher impact in the depressive-like offspring, which suggests that MS effects on cognition depend on the interaction of both genetic (depressive trait) and environmental factors (maternal care quality and early-life stress, here represented by MS), which may explain the smaller MS impact on cognitive performance of non-depressive-like offspring raised with more licking and grooming. It is also important to note that the learning deficits observed in Kyoto exposed to MS were more pronounced in females, while working memory deficits were more pronounced in males. Considering the major role of synaptic proteins in learning and memory, the observed higher vGlut1 expression in Kyoto adolescents, seems to be consistent with the memory deficits shown in the behavioral test. Our data highlight the relevance of vGlut1 in memory and reinforces previous studies [72]. On the other hand, Wistar adolescents presented increased BDNF in response to MS, which could had a protective role, preventing possible MS-induced learning deficits. Besides, high levels of licking and grooming have been associated with higher offspring BDNF levels [73], as such, the lack of this affiliative maternal behavior may contribute to explain the worse cognition observed in MS Kyoto adolescents.

Pro-inflammatory cytokines are critical factors for memory and cognition. Notably, IL-6 is important for spatial learning and reference memory formation [74, 75]. In agreement, the worse cognitive performance of the Kyoto adolescents was associated with reduced IL-6 expression. In agreement, the observed sex difference in learning in the Kyoto strain (MS Kyoto females presented a worse learning performance) was also associated with a decrease in IL-6. Further studies will be necessary to determine the nature of such effect. Adequate levels of IL-1β are required for learning and memory processes [76]. IL-1β expression decreased in the hippocampus of MS Kyoto adolescents, particularly in females, which performed poorly in the MWM. This is in accordance with previous studies, showing poorer performances in mice lacking the IL-1 receptor [45] or after the IL-1 receptor antagonist (IL-1ra) administration [77].

**Fig. 6:**
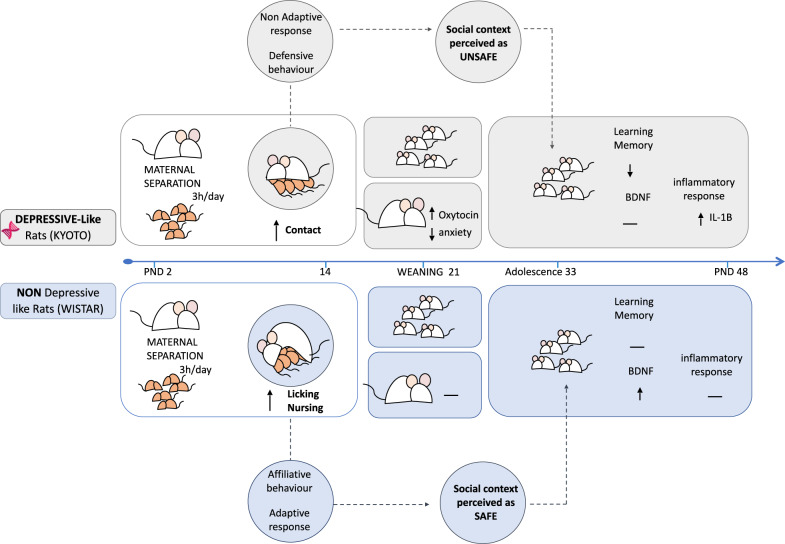
Graphical summary of main findings. Our results showed that mothers with different depression-like vulnerabilities facing maternal stress (induced by MS) adopted different strategies. Although both strains increased the maternal behavior in response to MS, non-depressive-like mothers (Wistar) exhibited a higher quality maternal behavior (affiliative), showing a more adaptive response and more pro-social behavior (more active strategy). On the other hand, depressive-like mothers (Kyoto) displayed a more passive/defensive strategy (non-adaptive response) in response to MS. The higher levels of oxytocin observed in Kyoto mothers that experienced MS seem to be part of the stress-response mechanism, which amplifies the negative perception of the environment (as insecure), leading to a more defensive behavior by keeping the pups very close to them and away from outsiders (out-group anti-social behavior). This increase in oxytocin expression seems to decrease the anxiety-like state in Kyoto dams in response to MS. Furthermore, the quality of maternal behavior observed in non-depressive-like mothers (Wistar) after MS seems to protect the cognitive performance of adolescent rats from the negative effects of MS, leading to an increase of BDNF expression in the offspring hippocampus. On the other hand, depressive-like adolescents showed lower resilience to MS effects, exhibiting worst performance in the cognitive tests and alteration in the IL-1β levels.

In summary, our study shows that beyond pro-social behaviors, oxytocin is involved in stress and mood disorder regulation (Fig. 6). We demonstrated that when a depressive mother is exposed to maternal stress, the release of endogenous hypothalamic oxytocin is triggered and leads to an altered perception of the environment (as insecure). Our study, in combination with previous studies [53, 54, 78], suggests that oxytocin not only increases sensitivity to environmental cues, but it may also amplify the social stress communication contributing to a social synchrony bidirectional intensification. This social synchrony in an adverse situation may contribute to better interactions, enabling the refinement of social communication and consequently promoting social cohesion as a defensive behavior toward potential threats. Finally, the different maternal stress-coping styles affect the offspring’s behavior, providing important clues about how early-life stress increases the risk for poor cognitive performances and how maternal care is a possible target for intervention.
